# Enabling Guidelines for the Adoption of eHealth Solutions: Scoping Review

**DOI:** 10.2196/21357

**Published:** 2021-04-30

**Authors:** Linn Nathalie Støme, Christian Ringnes Wilhelmsen, Kari Jorunn Kværner

**Affiliations:** 1 Centre for Connected Care Oslo University Hospital Oslo Norway

**Keywords:** eHealth, feasibility, global health, implementation

## Abstract

**Background:**

Globally, public health care is under increasing pressure, an economic burden currently amplified by the COVID-19 outbreak. With the recognition that universal health coverage improves the health of a population and reduces health inequalities, universal health coverage has been acknowledged as a priority goal. To meet the global needs in a population with increased chronic illness and longer life expectancy, the health care system is in dire need of new, emerging technologies. eHealth solutions as a method of delivery may have an impact on quality of care and health care costs. As such, it is important to study methods previously used to avoid suboptimal implementation and promote general guidelines to further develop eHealth solutions.

**Objective:**

This study aims to explore and thematically categorize a selected representation of early phase studies on eHealth technologies, focusing on papers that are under development or undergoing testing. Further, we want to assess enablers and barriers in terms of usability, scaling, and data management of eHealth implementation. The aim of this study to explore early development phase and feasibility studies was an intentional effort to provide applicable guidelines for evaluation at different stages of implementation.

**Methods:**

A structured search was performed in PubMed, MEDLINE, and Cochrane to identify and provide insight in current eHealth technology and methodology under development and gain insight in the future potential of eHealth technologies.

**Results:**

In total, 27 articles were included in this review. The clinical studies were categorized thematically by illness comparing 4 technology types deemed relevant: apps/web-based technology, sensor technology, virtual reality, and television. All eHealth assessment and implementation studies were categorized by their focus point: usability, scaling, or data management. Studies assessing the effect of eHealth were divided into feasibility studies, qualitative studies, and heuristic assessments. Studies focusing on usability (16/27) mainly addressed user involvement and learning curve in the adoption of eHealth, while the majority of scaling studies (6/27) focused on strategic and organizational aspects of upscaling eHealth solutions. Studies focusing on data management (5/27) addressed data processing and data sensitivity in adoption and diffusion of eHealth. Efficient processing of data in a secure manner, as well as user involvement and feedback, both throughout small studies and during upscaling, were the important enablers considered for successful implementation of eHealth.

**Conclusions:**

eHealth interventions have considerable potential to improve lifestyle changes and adherence to treatment recommendations. To promote efficient implementation and scaling, user involvement to promote user-friendliness, secure and adaptable data management, and strategical considerations needs to be addressed early in the development process. eHealth should be assessed during its development into health services. The wide variation in interventions and methodology makes comparison of the results challenging and calls for standardization of methods.

## Introduction

Health care constitutes a significant part of public sector expenditure. Total government expenditures among Organisation for Economic Co-operation and Development countries was 41% of the gross domestic product in 2015, and health typically accounts for around 20% of these expenditures [1]. There are several factors hindering efficient improvements in health care services across the globe; one of them is failure to adopt eHealth solutions [2]. With the recognition that universal health coverage improves the health of a population and reduces health inequalities, universal health coverage has been acknowledged as a priority goal of many health systems [3]. As such, the Commission on Social Determinants of Health emphasizes the importance of closing global and national health gaps through enhanced access to a high-quality and safe health supply [3]. As early as 1948, the United Nations declared enhanced access to a high-quality and safe health supply a priority goal [4]. To get around the logistical issues of delivering and receiving health care, both doctors and patients rely on digital communication channels to inform patients and enhance self-management.

Health interventions delivered suboptimally may reduce effective coverage [5]. Although life expectancy has increased globally following the United Nations declaration, the pace of improvement has been slowing down leaving behind large parts of the world’s population [6,7]. To achieve the ambitious task of universal health coverage by 2030 as postulated by the World Health Organization and World Bank, radical changes in social and economic trends are necessary [6,7], including improving daily living conditions; addressing inequitable distribution of power, money, and resources; and assessing the impact of action needed [8]. Successful implementation of eHealth solutions requires altering existing health care practices and therefore represents such an opportunity. New approaches delivered by eHealth solutions have the ability to enhance access and quality of care and reduce health care costs [9].

Digitalization, the process of transforming an organization into a digital form, may be considered a driving force in changing health care delivery and may improve accessibility globally [10]. In order to provide equal access to treatment of noncommunicable diseases, which represents the greatest health burden, the possibilities found within digitalization may be substantial [11]. Further, increase in life expectancy is expected to present an influx of patients health care systems are not equipped for. These patients commonly present with complex long-term needs requiring continuity of care, long-term hospitalization, and increased need for individualized care [12]. In addition, the recent COVID-19 pandemic has proved the importance of increased access to care while reducing risk of cross-contamination [13]. As such, several eHealth solutions have been rapidly put into use without a timeframe for tailored implementation or evaluation [14]. For health interventions to maximize benefit, they need to increase service quality and address the imminent needs of the target subpopulation: patients, health providers, and administrators [15].

A high-quality health service can only be achieved if patient outcomes and costs of delivery are addressed [16]. When considering complex health care systems, there are many areas of interest, including legal, organizational, economic, and social aspects, and each needs to be taken into account when looking to implement new technologies [17]. Within these areas, usability of product, learning curve for users, and the need for a recognized standard for patient data security have been highlighted as critical factors in eHealth implementation and scaling [18,19]. Adoption and diffusion of eHealth solutions may be time-consuming and require significant adaptation of work practices [20], and the development and rigorous evaluation of new models of care has been requested [21]. Research has found a discrepancy between the expected value of eHealth and mobile health interventions and the empirically demonstrated benefits [22,23].

In addition to evaluation of eHealth technologies, selection of correct implementation and scaling procedure should be prioritized. Varsi et al [24] found in a review of implementation strategies for eHealth that little attention has been paid to reporting implementation strategies for eHealth and that the feasibility may be compromised as the integration may be perceived as an interruption to existing patient workloads. Implementation processes may be complex as they may concern multilevel organizational structures and involve a wide range of health care stakeholders. A mixed method approach of assessment may be useful when preparing the health care sector for implementation. Applying quantitative methods to explore relationships between digital solutions and disease outcomes may prove useful in enhancing quality of care [25]. In addition, qualitative methods may provide a deeper understanding of contextual factors influencing these relationships and offer information on enablers and barriers to technological implementation globally [26]. This allows for iterative modifications and adaptations from the initial development phase to avoid implementation and scaling of ineffective services. To promote implementation of eHealth solutions with the greatest value to patients, the health care system, and society, we should look at potential value from the very start of the development cycle and throughout testing and implementation [27]. One challenge, however, is that early stages of innovation may suffer from lack of valid data sources.

This study aims to explore and thematically categorize a selected representation of early phase studies on eHealth technologies, focusing on papers that are under development or undergoing testing and assess the studies for common enablers and barriers of eHealth implementation. In summary, through exploring early development phase and feasibility studies we aim to provide applicable guidelines for evaluation throughout the stages of implementation.

## Methods

### Reporting Standards

The review was structured according to PRISMA (Preferred Reporting Items for Systematic Reviews and Meta-analyses) guidelines [28].

### Selection Criteria

Through two consecutive screenings, we reviewed the articles applying these inclusion criteria: (1) articles reporting some form of early stage eHealth solution (articles were excluded if the intervention or innovation did not consist of eHealth solutions) and (2) articles presenting particular focus on mixed methods to evaluate the feasibility of eHealth (articles were excluded if they did not evaluate the feasibility of eHealth solutions). Detailed inclusion and exclusion criteria can be seen in Textbox 1.

**Selection criteria.**

Inclusion criteria:

- Study type
  Early phase qualitative and mixed method studies
  Reviews
- Technology (population)
  eHealth
  mobile health
- Intervention
  Apps
  Sensor technology
  Virtual reality
  Television
- Outcome
  Technological solutions/health care services
  Methods of evaluation
  Implementation of eHealth solutions as opposed to specific technologies

Exclusion criteria:

- Study type
  Language not in English, Norwegian, or Danish
  Purely quantitative research
  Published prior to 2008
- Technology (population)
  No digital innovations
  Articles deemed not innovative enough
- Intervention
  Short message (texting) services
  Interventions not containing digital innovation
- Outcome
  Articles not falling into categories stated in the inclusion criteria

### Outcome Measures

This study aims to explore eHealth technologies under development or undergoing testing to identify enablers and barriers to implementation. We used thematic analysis to categorize the studies according to a preselected thematic framework comprising the following categories: user, technology, analysis, country of origin, and focus point.

### Search Strategy

A literature search was conducted in April 2019 using PubMed, Ovid MEDLINE, and Cochrane. The specifics of the search were constructed and performed with the assistance of a librarian with experience in systematic search methods. The search was done using a variety of text words and subject headings. The search strategy for MEDLINE was built using the MeSH terms “telemedicine,” “home care services,” “self-help devices,” “communication aids for disabled,” “information technology,” “biomedical technology,” or “telenursing” and synonyms and near-synonyms thereof combined with the text words “technology assessment, biomedical.” The full search strategy can be found in Multimedia Appendix 1.

### Selection of Studies

Textbox 1 shows the final inclusion and exclusion criteria agreed upon by the review group. Due to the high number of articles found through the initial search, certain parts of the inclusion criteria were added during the elimination process, mainly in the Intervention category. In addition, we excluded papers published before 2008 partly due to relevance but also to decrease the number of articles. References from each database search were imported into database-specific folders in EndNote (version X9, Clarivate Analytics), and duplicates were eliminated. Abstracts were first assessed by CRW using the selection criteria listed in Textbox 1, and then each of the full-text articles was appraised independently by two reviewers (CRW and LNS). Disagreements were resolved by discussion or by referring to a third author (KJK).

The topic of eHealth solutions is rather broad, and it is challenging to ensure inclusion of all relevant papers within this topic. In an effort to thematically analyze the included studies, the studies were initially thematically categorized in disease groups that represent a great health burden and cost for society, including cardiovascular diseases, cancer, diabetes, and chronic lung disease, as they make the largest contribution to morbidity and mortality [29]. Regarding technology, we focused on commonly used eHealth solutions. However, as the aim of this scoping review was to study barriers and enablers of eHealth implementation, some studies were included as they either focused on specific barriers or enablers of eHealth implementation or evaluated promising technologies less widespread.

The data were extracted by CRW and discussed with LNS. During this process, a framework based on the assessed literature was agreed upon and core themes to answer the research issue were identified. When there was a disagreement among the authors on the appropriate theme, the article was discussed until agreement was achieved. Bibliographic data and study content were collected and analyzed using templates developed iteratively with feedback from the other authors (KJK and LNS).

### Data Synthesis and Analyses

Data from the included studies were categorized in Table 1 to provide an overview of the study characteristics for further assessment. Through this categorization and subsequent analysis, we aimed to study barriers and enablers to eHealth implementation.

The data extracted from the included studies were categorized based on Støme et al [30] as follows:

User: Data were categorized by user group targeted by the study: chronic obstructive pulmonary disease (COPD)/asthma, cardiovascular disease, diabetes, elderly adults, and other/not specified.

Technology: Studies were categorized as clinical if a specific technology was applied and divided into four subgroups: apps/web-based technology, sensor technology, virtual reality, and television. Studies were categorized as theoretical if eHealth implementation issues were addressed and divided into the subgroups usability, scaling, and data management.

Analysis: Identified articles were characterized by strategic, economic, and clinical analysis based on the purpose of the analysis and not the analytical approach used, as one analytical approach can be used for different purposes.

Country: Original articles were categorized by country of origin. Reviews were categorized by country of publication or origin of authors. Case studies were categorized by country where study took place.

Focus point: Studies addressing eHealth assessment and implementation were categorized by usability, scaling and data management.

## Results

### Study Selection

An initial search identified 18,613 studies, and reviewers performed a rough quality assessment by searching through EndNote for the terms “eHealth,” “implementation,” “feasibility,” “early phase” to explore and thematically categorize early phase studies on eHealth technologies under development or undergoing testing. The coarseness of this method was deemed necessary due to the large amount of papers following the initial search. The primary search also included home-hospital services, which did not include eHealth solutions. In the selection of relevant studies for this scoping review, we therefore excluded a large number of studies of moving care to the patients’ home without the use of digitalization or eHealth solutions. Following this method, 628 papers remained. To further narrow the scope, CRW assessed the abstract texts for relevance to the topic, including studies on early phase eHealth development and adoption. Papers published prior to 2008 were excluded as we deemed this to be a suitable breakpoint for technological progress, and articles published prior to this year could have been outdated. Due to the rapid progression of technology development, at a certain point eHealth solutions become outdated when new technology enters the market. Although 2008 is not a year specific for such change, we deemed it adequate to avoid evaluating outdated technology. Following the second assessment, 81 articles remained.

**Figure 1:**
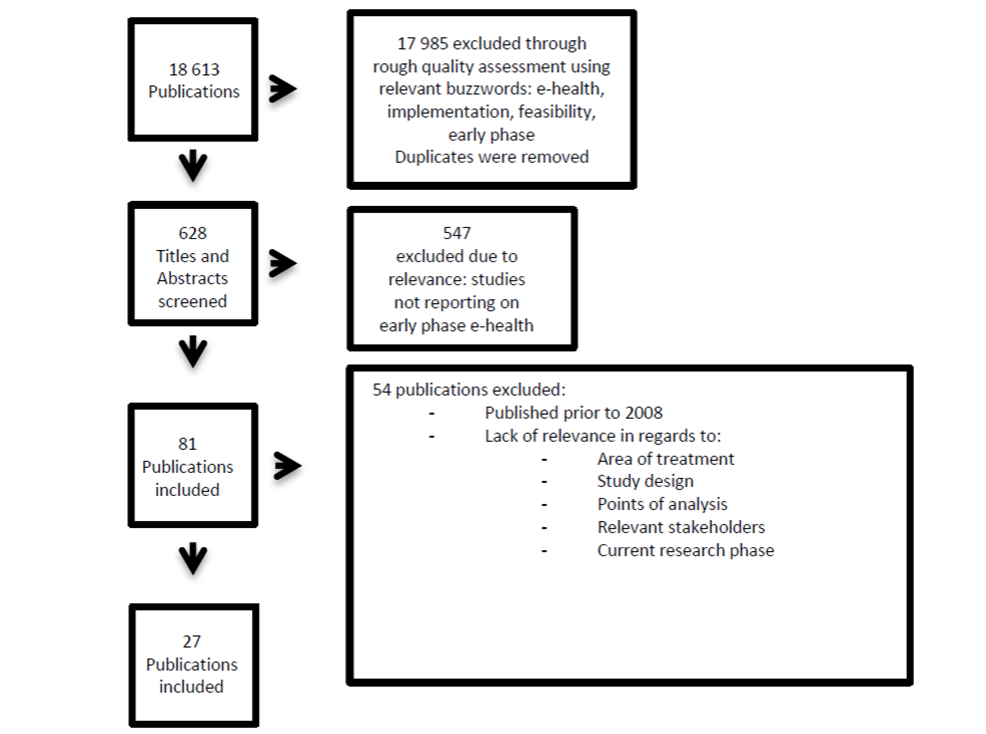
Flow diagram for studies included in the review.

The studies were rigorously assessed, entered on a spreadsheet, and given a general rating based on study design and points of analysis. Relevant stakeholders, advantages of the approach, current research phase, and situational value of the technology were also considered. A focus on variance between papers to gain a holistic view of the topic was an underlying factor for selection. Following this assessment, 27 reviews, pilot studies, and other forms of research papers were chosen as the basis for this review. The underlying intention behind this review was to identify works of literature and articles addressing both methodological approaches and eHealth solutions in their early phase/concept development/pilot studies. Black et al [22] observed a gap in methodological approaches to studying the empirical evidence concerning the effect of eHealth interventions. Through our search it became apparent that few articles capture implementation and assessment of eHealth. The papers in our review focus on technologies used to treat chronic conditions. An overview of the study selection is shown in Figure 1.

### Study Characteristics

The studies selected to undergo a thorough evaluation are shown in Table 1. Enablers and barriers of eHealth implementations will be discussed further in light of findings from these studies. Based on their main focus point, the studies were divided into the categories usability, scaling, or data management. Studies focusing on usability (16/27) mainly addressed user involvement and learning curve in the adoption of eHealth, while the scaling studies (6/27) focused on strategic and organizational aspects of upscaling eHealth solutions. Studies focusing on data management (5/27) addressed data processing sets and data sensitivity in adoption and diffusion of eHealth.

The studies were thematically analyzed and sorted into 5 categories based on illness, although some degree of overlap was found in some studies: patients with COPD/asthma (6/27) [31-35], patients with cardiovascular disease (3/27) [36-38], patients with diabetes (2/27) [39,40], and elderly patients (8/27) [41-48]. While the majority of apps/web-based technology had disease-specific targets, the other technologies were used more frequently with elderly patients. The remaining 8 studies were categorized as other or nonspecified, as illness was not described [49-55] or they focused on issues more relevant from an organizational or caregivers point of view [56,57]. These studies provided theoretical insight into how a given technology may assist in remote treatment and care [49-57].

The majority of the studies were from western countries: 10 studies originated from the United States [33,37,39,44,47-49,51,56,57], 3 from Australia [31,41,55], 4 from England [32,36,45,53], and 3 from Italy [42,43,46]. The remaining 7 studies each took place in Belgium [40], Denmark [35], Japan [34], Malaysia [53], Netherlands [38], Switzerland [54], and Norway [50], respectively.

**Table 1:** Description of the data and data analysis.

Author (year)	User	Technology	Focus point	Analysis	Country
Abd Sukor et al (2015) [41]	Elderly adults	Data algorithm	Data management	Clinical analysis	Australia
Aguilar et al (2014) [49]	Not specified	Multifaceted	Scaling	Strategic analysis	US^a^
Angius et al (2008) [42]	Elderly adults	Television	Usability	Strategic/economic analysis	Italy
Arlati et al (2019) [43]	Elderly adults	Virtual reality	Usability	Strategic analysis	Italy
Bartels et al (2017) [44]	Elderly adults	Multifaceted	Scaling	Strategic analysis	US
Burkow et al (2008) [50]	Unspecified chronic disease	Television	Usability	Strategic analysis	Norway
Burrige et al (2017) [36]	Cardiovascular disease	Sensor technology	Usability	Clinical/strategic analysis	England
Cai et al (2015) [45]	Elderly adults	Sensor technology	Usability	Strategic analysis	England
Chi and Demiris (2015) [56]	Chronic disease patients’ caregivers	Multifaceted	Usability	Strategic analysis	Primarily US
Ding et al (2012) [31]	COPD^b^	App and web portal	Data management	Clinical/strategic analysis	Australia
Fitzsimmons et al (2016) [32]	COPD	Sensor technology	Usability	Strategic analysis	England
Georgsson and Staggers (2017) [39]	Diabetes	App and web portal	Scaling	Strategic analysis	US
Hui et al (2017) [33]	COPD	App	Usability	Clinical analysis	US
Kamei et al (2012) [34]	COPD	Sensor technology	Usability	Economic/clinical analysis	Japan
Kitsiou et al (2015) [37]	Cardiovascular disease	Multifaceted	Scaling	Strategic analysis	US
Lilhot et al (2015) [35]	COPD	Web-based and tablet technology	Usability	Strategic analysis	Denmark
Mantas et al (2009) [57]	Data security	Data processing algorithm	Data management	Strategic analysis	US
McLean et al (2013) [51]	Not specified	Multifaceted	Scaling	Clinical/economic analysis	US
Monkman (2015) [38]	Cardiovascular disease	App	Usability	Strategic analysis	Netherlands
Mora et al (2018) [46]	Elderly adults	Sensor technology	Data management	Strategic analysis	Italy
Norman et al (2018) [47]	Elderly adults	Multifaceted	Usability	Strategic analysis	US
Sanders et al (2012) [52]	Not specified	Multifaceted	Usability	Strategic analysis	England
Simon and Seldon (2012) [53]	Not specified	App and sensor technology	Data management	Strategic analysis	Malaysia
Tschanz et al (2017) [54]	Not specified	App	Usability	Strategic analysis	Switzerland
Hoecke et al (2010) [40]	Diabetes	App	Usability	Strategic analysis	Belgium
Wade et al (2016) [55]	Not specified	Multifaceted	Scaling	Strategic analysis	Australia
Weiner et al (2016) [48]	Elderly adults	Virtual reality	Usability	Strategic analysis	US

^a^US: United States.

^b^COPD: chronic obstructive pulmonary disease.

### Intervention Characteristics

The characteristics of the interventions by technology and illness are summarized in Table 2. The categories were determined following the selection of studies, in an attempt to categorize the studies. The studies were divided into 5 categories based on the type of platform they used, although overlap between technologies was shown in some studies. Among the clinical studies, 8 presented apps combined with web-based technology as their intervention [31,33,35,38-40,53,54]. This was the most common study technology, mainly applied in patients with COPD and asthma. Five studies used sensor intervention, located on the human body or placed indoors in the patients’ home [32,34,36,45,46]. Two studies used virtual reality as a method of intervention [43,48]; in the remaining 2, television was tested as a suitable technology [42,50].

Ten studies were categorized theoretical as they did not present a particular technology but focused on general remote care issues [37,41,44,47,49,51,52,55-57]. The studies were placed in one of the 3 subcategories: usability [47,52,56], scaling [37,44,49,51,55], and data management [41,57], depending on their primary point of focus. Of these, the majority had communication between patient and practitioner as their primary goal.

**Table 2:** Study categorization.

Technology/remote care issues	Clinical studies				Theoretical studies				Total
	Apps/web-based technology	Sensor technology	Virtual reality	Television	Usability	Scaling	Data management		
COPD^a^/asthma	4	2	—^b^	—	—	—	—	6	
Cardiovascular disease	1	1	—	—	—	1	—	3	
Diabetes	2	—	—	—	—	—	—	2	
Elderly adults	—	2	2	1	1	1	1	8	
Other/not specified	1	—	—	1	2	3	1	8	
Total	8	5	2	2	3	5	2	27	

^a^COPD: chronic obstructive pulmonary disease.

^b^Not applicable.

### Assessment of eHealth Solutions

Early assessment of the potential effect of eHealth solutions was studied in this scoping review with the aim of providing guidelines for evaluation at different stages of implementation. Feasibility can be assessed through qualitative and quantitative methods and may reflect strategic analysis to prepare for implementation or show clinical and safety aspects of the care provided. The feasibility of eHealth interventions was assessed in 6 studies [31,36,41,43,46,50] applying acceptability, usability, and/or utility data from the intervention. Qualitative assessments of the effects of eHealth solutions were also found in this review [32,39,45,47,48,52,54,55]. A Cochrane review [58] identified the need for additional qualitative research to determine if and why particular eHealth interventions are effective. Fitzsimmons et al [32] observed a need to integrate patient satisfaction measures based on patient perceptions of the eHealth technology.

In this review, 2 studies reported on heuristic approaches to evaluation of eHealth solutions [35,38]. These studies emphasized the need to enhance usability in the development and testing of new eHealth solutions. Usability is the extent to which a product or service may be used by a cohort of users to achieve effectiveness, efficacy, and satisfaction in a specified context of use [59]. This may be enhanced by studying users and behavioral data collected during the development of technologies [32]. Such assessments may be time-consuming and resource demanding. Heuristic methods represent a timelier and less expensive approach to assess usability, as user friendliness may be a requirement to achieve full benefit from eHealth solutions in terms of clinical outcomes and patient satisfaction [35].

## Discussion

### Principal Findings or Summary

This scoping review identified apps/web-based technology and sensor technology as commonly used thematic technologies. Virtual reality, while not as commonly used within eHealth solutions, showed promising results in the studies identified and was included in this study in part due to its wide potential. Television was also found to be used to a somewhat lesser extent, but we included it in this paper due to ease-of-use and high accessibility across patient groups. Within the representative studies assessed in this study, apps/web-based technology had disease-specific targets, while the other technologies were primarily used in elderly patients, where the desire for individualized and tailored care is high [60]. Through analyzing the studies, we identified usability, scaling, and data management as important research areas regardless of eHealth implementation. Studies focusing on usability mainly addressed user involvement and learning curve in the adoption of eHealth, while the majority of scaling studies focused on strategic and organizational aspects of upscaling eHealth solutions. Studies focusing on data management addressed data processing and data sensitivity in adoption and diffusion of eHealth. To explore factors critical for implementation of eHealth based on the studies included in this review, data security and processing, user involvement and feedback, and transitioning from small- to large-scale implementation will be discussed below.

We found that although the emergence of eHealth technologies creates a plethora of innovation opportunities, it is apparent that proper guidelines for evaluating sufficient quality of product are currently not available. This may result in a lack of implementation of new technologies [26,40,43]. As the aim of this study was to explore early development phase and feasibility studies as an intentional effort to provide applicable guidelines for evaluation at different stages of implementation, contributions found in the literature are discussed in this section in the three focus areas: usability, scaling, and data management.

Today, people-centered health care is an increasing ambition [61], and health care is moving toward reduced hospital stays and emphasis on technology-driven solutions to support arena-flexible treatment strategies. Correspondingly, the engagement of end users has become a necessary component in the design and development of future health care [61]. Emphasis is on evidence and outcomes, and the participation of users in the provision of their own care has become essential [48]. User-centric design involving patients and health care providers can be employed from the earliest exploratory stages to help understand and design for the needs, goals, limitations, capabilities, and preferences of all stakeholders [62]. Technological development is constantly evolving, and continuous technological adoptions are challenging the identification of valid outcome measurements suitable for assessment of cost and patient benefits [63]. A potential solution may be to integrate an assessment of the whole development cycle, in order to help identify shortcomings and suboptimal parts/areas of innovations. The earlier stages of the development cycle, such as concept stages of innovation, may, however, suffer from lack of valid data sources. This may explain the heterogeneity in the evidence concerning the effect of eHealth interventions in the literature [22,64,65].

### Data Management in the Integration of eHealth

Efficient data transfer between parties without compromising the security of sensitive data is important to account for when integrating eHealth solutions. There are vast numbers of requirements for high-security protocols in eHealth. This is obviously due to the sensitive, and in some cases vital, nature of the data and should not be taken lightly [57]. Due to the large amount of data cryptically transferred from patients to practitioners and vice versa, suitable processing technology needs to be incorporated [41]. Especially in the cases of self-measurements, the amount of data is often vast and diluted with errors and noise artefacts since patients are often unaware of what they are supposed to look for [41]. Suitable systems for data processing should therefore be incorporated from an early phase to support the intent of lessening the workload through eHealth [41]. Only through implementing optimal security and processing programs into eHealth can an integrated approach to health care be achieved [27,49]. A summary of guidelines concerning data management in the integration of eHealth can be found in Table 3.

**Table 3:** Summary of guidelines for eHealth implementation.

Guidelines	Reference	Summary
Data management	[27,41,49,57]	Efficient data transfer between parties without compromising the security of sensitive data. Only through implementing optimal security and processing programs into eHealth can an integrated approach to health care be achieved.
User adaptations	[29,32,39,44,45,66-68]	User involvement may enhance usability and is a significant factor in the implementation of eHealth. The need to account for patient and practitioner adherence was a common feature the reviewed articles reported on. User training programs must provide such information to enhance self-management and goal achievement.
Evaluation and scaling	[23,32-36,38,47,55]	Four critical barriers affecting providers and patients in clinical implementation of eHealth are reported: technological illiteracy and lack of knowledge, awareness, and access to the technology itself. Early stage evaluations of eHealth may reveal hidden factors for successful implementation. Integration of eHealth interventions must be seen as part of a service and not as a standalone system. Two key actions for sustainable implementation are the marketing of eHealth to patients, clinicians, and policymakers and establishing a practice community.

### User Adaptations to eHealth Solutions

User involvement may enhance usability and is a significant factor in the implementation of eHealth [66-68]. In the articles reviewed that reported on patient and practitioner feedback, several key points were highlighted and should be accounted for whenever a technological innovation is evaluated. A common feature the reviewed articles reported on was the need to account for patient adherence [29,39]. However, the patients need to understand what to do, why they do it, and how the eHealth solution works to adhere to its use [44]. User training programs must provide such information to enhance self-management and goal achievement [39]. Rigorous training programs may be needed to facilitate successful self-management of the technology [39], as patients vary in understanding and personal motivation. Self-management helps patients gain a better understanding of their condition and enables better communication with their practitioners. This may also ease the intended transparency between patients and practitioners.

Rigorous training may not be enough to ensure uptake of eHealth solutions, and active user involvement in the design of eHealth solutions needs to be perceived as valuable for the participants, such as health care providers. To ensure successful implementation of eHealth, practitioner adherence is also required. Cai et al [45] reported that practitioners involved in the introduction of the technology gain a better sense of the value of the technology they are applying. Studies also highlight positive feedback from patients when the technology facilitates an interactive relationship between patient and practitioner, such as activity planning and communication with practitioners or other health care staff [32]. In this, however, it is increasingly important to uphold a robust level of data security and privacy [69], another factor to be thorough about throughout implementation of any technology [45]. A summary of guidelines concerning user adaptations of eHealth solutions can be found in Table 3.

### Evaluation and Scaling of eHealth Solutions

Successful implementation of eHealth solutions may require altering existing health care practices, which may influence patient-provider relationships. Four critical barriers affecting providers and patients in clinical implementation of eHealth are reported: technological illiteracy and lack of knowledge, awareness, and access to the technology itself [32,34]. As these barriers will vary greatly depending on social, geographical, and individual situations for patients and caregivers, innovators need to be aware of and make room for individualized alternatives within a given solution. In other words, optional customization within a given eHealth solution to account for each scenario should be included. Timely implementation of eHealth solutions is challenged by lack of early indications of patient benefits and costs. The purpose of this study was to explore how early assessment of eHealth solutions is communicated in the literature to study which markers of eHealth performance could be detected in an early phase. To ensure effective implementation and diffusion of eHealth solutions, each of these barriers needs to be addressed and assessed during the development process of the solution [36]. Early introduction and evaluation of the technology under development is therefore critical. Adaptations to the intervention are still possible in this stage, and barriers to implementation may be identified and eliminated. Early stage evaluations of eHealth may reveal hidden factors for successful implementation. To maximize the benefits associated with eHealth interventions while minimizing risks, evaluations of eHealth interventions should be performed during both design and deployment [70].

Heuristics are decision-making methods that may be applied when faced with short time frames and lack of resources with which to analyze complex data. Although heuristics may be helpful in many situations, the use may also lead to bias, as decisions made using a heuristic approach are likely to be suboptimal [35,38]. As such, to ensure the right eHealth solutions are adopted, increasing the pace of evaluations of eHealth solutions must not sacrifice the quality of scientific findings. Munafò et al [71] call for increased reproducible science and the need to implement measures to improve research efficiency and robustness of scientific findings. The authors argue for the adoption, evaluation, and ongoing improvement of measures to optimize the pace and efficiency of knowledge accumulation.

Evaluating eHealth technologies means evaluating the health care service as a whole. In other words, integration of eHealth interventions must be seen as part of a service and not as a standalone system. eHealth is designed to support the relationship between patients and their health care providers and will never replace the personal interaction between patient and provider [72]. This is why successful implementation requires a holistic approach including the technology, organizational structures, change management, economic feasibility, societal impacts, perceptions, user-friendliness, evaluation and evidence, legislation, policy, and governance [73].

Positive outcomes of any given technology implemented in health care will need to undergo upscaling to suit its intended use and maximize potential benefits. Wade et al [55] developed a qualitative framework for executing large-scale implementation of eHealth solutions. To produce an approach to implementation that ensures sustainable adoption in clinical environments, two key actions were highlighted: marketing of eHealth to patients, clinicians, and policy makers and establishing a practice community. Such leadership support may be vital to large-scale implementation. Policy makers also need awareness of how eHealth aligns with health care policies and how evidence of functionality may best be demonstrated to clinicians [55].

We found large diversity in the studies on the effect of eHealth. To influence policy makers’ and clinicians’ interpretation of outcomes, research is proposed to be dedicated to understanding optimal strategies for implementation [23,33]. Policy makers and local decision makers may need to adjust their expectations of immediate clinical or economic benefits of eHealth, as it is suggested that the greatest gains may be achieved for patients at highest risk of serious outcomes [47]. A summary of guidelines concerning user adaptations of eHealth solutions can be found in Table 3.

### Comparison With Prior Work

Through analysis of the relevant literature we identified several systematic reviews conducted by other research groups. While some of these studies addressed different stages of implementation, others highlighted specific factors of eHealth applications, patient subgroups, or diseases as their focus. Chi et al [56] focused on eHealth experience and innovative potential for patients’ caregivers. While this study presented valuable insight into the application of eHealth, it did not provide a full picture of the subsequent effects of eHealth technology. Kitsiou et al [37] provided a broad overview by analyzing several systematic reviews but focused its efforts on patients with chronic heart failure.

Ekeland and Linstad [74] provided valuable insight to different models of eHealth governance but did not give insight into how different technologies may be received by their user groups. Schreiweis et al [75] studied enablers and barriers to eHealth implementation and presented similar conclusions as this study. The literature analysis presents expert discussions to emphasize their findings on enablers and barriers, while this study presents findings on evaluations methods applied in early assessment of eHealth. As such, this study explores the need for early evaluation to communicate the innovative potential in future eHealth research. Ross et al [19] studied eHealth implementation in a comprehensive review of reviews of eHealth and found that a frequent reason for unsuccessful implementation is that the information systems do not fit well with work practices or daily clinical work. Similar to this study, the authors also emphasize the need to focus on reflecting and evaluating the potential benefit of eHealth solutions. For software evaluations, the International Organization for Standardization/International Electrotechnical Commission (ISO/IEC) has defined evaluation methods for the quality of software products and provided common standards called the Systems and Software Quality Requirements and Evaluation series including ISO/IEC 25022:2016 and ISO/IEC 25023:2016 [76]. This quality evaluation framework focuses on metrics such as functional suitability, reliability, performance efficiency, usability, security, compatibility, maintainability, and portability, which are essential to ensure robust eHealth solutions. However, as emphasized above, integration of eHealth interventions must be seen as part of a service and not as a standalone system. As such, there is a need to establish an agreed upon evaluations framework to support eHealth solutions throughout the life cycle.

While the previous and related studies show similarities to this study, the focus of this study on the early development phase provides unique insight into factors important to consider when implementing eHealth solutions. Through exploring the early development phase and feasibility studies, this study seeks to provide the groundwork for applicable guidelines for early evaluation of eHealth solutions. As such, this scoping review may be applied as a roadmap to future studies.

### Limitations

This scoping review may not have identified all published studies on the feasibility of eHealth, in particular the grey literature. The search strategy may have been compromised by nonstandardization of vocabulary in this relatively new field of research. As the literature search was conducted in two iterations, studies on the feasibility of eHealth may have been involuntarily excluded. As such, the representativeness of the selection of studies evaluating the feasibility of eHealth solutions may have been compromised. In addition, the attempt to draw out representative studies may have inadvertently caused this study to provide a small snapshot of the broad picture that is eHealth implementation. The results from a subset of this small scale may therefore be more fragile to potential outliers, changes in protocol, and new findings emerging in the coming years, as well as findings from the few years since the search was conducted in 2019. Using MeSH terms does not include non-MEDLINE indexed journals, which is a significant subset of PubMed-indexed papers. As such, JMIR Res Protoc or JMIR Formative Res publications were not included in this search, as they were not MEDLINE-indexed 2 years ago. This constitutes a major limitation to the search strategy in this paper. This scoping review did not include specific evaluation frameworks for eHealth components such as software quality but rather focused on evaluation of eHealth as an integrated part of a health system. Despite attempts to adjust the search strategy to several different terms previously used in the literature to describe similar methodologies, other terms may also exist. Although three comprehensive health databases were included in the search (PubMed, Ovid MEDLINE, and Cochrane), searching other databases may have included additional published studies. Our search included only studies in English, Norwegian, and Danish, although only English terms were used in the search. Furthermore, no consultations with stakeholders or experts were included in this review. Finally, although the method was systematically followed by the reviewers, each reviewer subjectively included studies based on the study criteria. Reviewer bias may have occurred in the attempt to include studies that represent a holistic view of eHealth solutions under development and testing. The classification and interpretation of the results were also subject to reviewer bias.

### Further Research

As technology continually advances, so does the number of eHealth solutions. Additionally, its infusion into health services is emerging as an active area of research. This was seen in several studies in our literature search. The diversity of studies demonstrated that eHealth is a continually developing field. A large heterogeneity in methodology, sample, interventions, processes, and outcomes was found. It gives an overview of the current broad spectrum of methods but also reflects the broad eHealth scope: to improve health care and enhance quality of care.

Patients also represent diversity, as each of us is different and solutions need to be tailored to the individual. It might also mean that for more robust conclusions to be drawn, improvement related to methodology and standardization is needed. Several studies in this review were not free of bias, reported lack of blinding and related outcomes to the Hawthorne effect. Before a standardized recommendation for methodology concerning assessment and implementation of eHealth can be finalized, more research is needed to systematically validate the methods used for evaluating and implementing eHealth technologies. However, while standardization of methods can achieve better streamlining of new technologies, it is important to keep in mind that diversity and innovation within methodologies can also lead to improvement of innovative methods. Standardization of methodology, with sequential adaptations to new practices, may be a suitable way to optimize eHealth implementation methods. One approach for determining best practice may be to conduct mini case studies on the different methods of implementation and potential subsequent merging methods.

### Conclusion

In conclusion, eHealth interventions have considerable potential to improve lifestyle changes and adherence to treatment recommendations, at least in the short term. While apps may support patients with self-management and increased adherence to treatment recommendations, sensor technology may provide additional use and data generation in the health care sector. Virtual reality has a role as a tool to support patient engagement, as well as providing a social platform for isolated patients. The use of television as a medium for system design may help alleviate barriers to user friendliness, as it has been a common household accessory for a long time. However, individualization, data management, and user-friendliness are important factors for use, and technical challenges need to be overcome for full integration to succeed. In terms of providing guidelines for evaluation at different stages of implementation, we found that usability, data management, and scaling strategies should be enhanced in early stage evaluations of future eHealth solutions. Evaluating an eHealth solution still under development may provide continuous information on the performance of the intervention in different development and pilot stages. As such, ineffective solutions may be rejected at an early stage, making room for innovations that provide the most benefit for society. The wide variation in interventions and methodology makes comparison of the results challenging and calls for standardization of methods. A stepwise approach by using subgroup analysis may be one solution that may allow us to understand patient characteristics, behavior, needs, and preferences, allowing us to tailor interventions to those patterns and achieve improved health outcomes while reducing costs. Follow-up, long-term interventions, and analysis of cost-effectiveness need to be included in future research.

eHealth has the potential for refinement and personalization of existing health care practices and may be of great value. However, user involvement, training, and scaling strategies are important features to implement from the initiation of the development process.

## Appendix

Multimedia Appendix 1 Search strategy Ovid MEDLINE.

## Abbreviations

COPD: chronic obstructive pulmonary disease
IEC: International Electrotechnical Commission
ISO: International Organization for Standardization
PRISMA: Preferred Reporting Items for Systematic Reviews and Meta-analyses
