# Supplementary material for: Enabling Guidelines for the Adoption of eHealth Solutions: Scoping Review
Source: JMIR Form Res. 2021 Apr 30;5(4):e21357. doi: 10.2196/21357 (PMC8122291; doi:10.2196/21357)
Supplement: Multimedia Appendix 1 [file formative_v5i4e21357_app1.doc]

# Multimedia Appendix 1

Database: Ovid MEDLINE(R) ALL <1946 to April 02, 2019>

Search Strategy:

--------------------------------------------------------------------------------

1 telerehabilitation/ (230)

2 (Telebehavioral or Tele-behavioral).mp. (24)

3 (telehome* or tele-home* or telerehab* or tele-rehab* or telecare or tele-care or telemonitoring or tele-monitoring).ti. (1605)

4 welfare technolog*.ti. (9)

5 (ambient and assist* and living).ti. (79)

6 (smarthome* or smartcare or smart care or smart environment* or (smart and (technolog* or home* or house*))).ti. (551)

7 home technolog*.ti. (30)

8 home health technolog*.ti. (4)

9 ((home* or remote or distance) and rehab* and technolog*).ti. (19)

10 ((home* or remote or distance) and monitor* and (patient* or technolog*)).ti. (707)

11 ((home* or remote or distance) and medical technolog*).ti. (17)

12 ((remote or distance) and (consult* or counsel* or communication*) and patient*).ti. (30)

13 or/1-12 (3117)

14 (home* or telehome* or domest* or domicil* or dwell* or community-living or independent living or independently living).ti. (152796)

15 (self-monitoring or self-care or self-management or self-medication or self-administration).ti. not medline.st. (2387)

16 (outpatient* or ambulatory).ti. not medline.st. (4947)

17 (Aging in place or ageing in place or age in place).ti. not medline.st. (42)

18 independent living/ (4068)

19 homebound persons/ (585)

20 *community health services/ or *community health nursing/ or home health nursing/ or distance counseling/ or exp home care services/ (74920)

21 exp *Self Care/ (24031)

22 Self-Management/ (1036)

23 *Ambulatory Care/ (18592)

24 *Outpatients/ (5177)

25 exp *Monitoring, Ambulatory/ (12421)

26 *Rehabilitation/ or *Cardiac rehabilitation/ or *Activities of daily living/ or *Neurological rehabilitation/ or *Stroke rehabilitation/ (43009)

27 *Self-help devices/ or *Communication aids for disabled/ (4823)

28 *Chronic Disease/ (24214)

29 *Disabled Persons/rh [Rehabilitation] (3484)

30 *"delivery of health care"/ or *health services accessibility/ (86818)

31 *"continuity of patient care"/ or *patient discharge/ (21097)

32 *adolescent health services/ or health services for persons with disabilities/ or *health services for the aged/ (17253)

33 exp *Health Services Research/ (50980)

34 *Health Services/ (15881)

35 or/14-34 (500706)

36 *Technology/ (4994)

37 *Biomedical Technology/ (4158)

38 Information Technology/ (193)

39 *telecommunications/ or *electronic mail/ or telemedicine/ or remote consultation/ or *telemetry/ or *remote sensing technology/ or *telephone/ or *cell phone/ or *text messaging/ or *videoconferencing/ or *wireless technology/ or distance counseling/ (45263)

40 *computing methodologies/ or *artificial intelligence/ or *robotics/ or *virtual reality/ or *internet/ or *internet access/ or *social media/ or *computers/ or *microcomputers/ or *computers, handheld/ or *smartphone/ or *software/ or mobile applications/ or *software design/ or *speech recognition software/ or *user-computer interface/ (149736)

41 *Hotlines/ (1709)

42 *Attitude to Computers/ (2112)

43 Telenursing/ (199)

44 *Computer-Assisted Instruction/ (8696)

45 *Reminder Systems/ (1947)

46 *Geographic Information Systems/ (2940)

47 "Cell Phone Use"/ (80)

48 *Wearable Electronic Devices/ (661)

49 Consumer Health Informatics/ (63)

50 *Technology Assessment, Biomedical/ (5568)

51 (ehealth or ehealth or m-health or mhealth or e-medicine or emedicine or e-therap* or etherap* or e-consult* or econsult* or e-counsel* or ecounsel* or e-based or ebased or e-care or ecare or mcare or m-care).ti. (2568)

52 (telemedical or telemedicine or telecommunication* or telehealth* or tele-health* or telenurs* or tele-nurs* or telemetr* or teleconsult* or tele-consult* or tele-counsel* or telecounsel* or telebehavior* or tele-behavior*).ti. (10761)

53 (mobile health or (mobile and (technolog* or phone* or application*))).ti. not medline.st. (1536)

54 (app or apps).ti. not medline.st. (1752)

55 (cellphone* or smartphone* or smart phone*).ti. not medline.st. (1403)

56 internet.ti. not medline.st. (2310)

57 remote monitoring.ti. (584)

58 health technolog*.ti. not medline.st. (398)

59 healthcare technolog*.ti. not medline.st. (17)

60 (digital or interactive or interactivity or online or on-line or web or website* or tablet or tablets or e-mail or email or "electronic mail" or "text messaging" or sms or "short message service" or software).ti. not medline.st. (21145)

61 or/36-60 (245696)

62 13 or (35 and 61) (18629)

***************************
